# Incubation Time and Size Effects of Biodegradable Mulch Microplastics on Lettuce Plantlets In Vitro

**DOI:** 10.3390/plants15050849

**Published:** 2026-03-09

**Authors:** Mathilde Henrion, Lluis Martin-Closas, Iseult Lynch, Ana M. Pelacho

**Affiliations:** 1 Department Agricultural and Forest Science and Engineering, University of Lleida, 25198 Lleida, Spain; mathilde.henrion@udl.cat (M.H.); lluis.martin@udl.cat (L.M.-C.); 2 School of Geography, Earth and Environmental Sciences, University of Birmingham, Birmingham B15 2TT, UK; i.lynch@bham.ac.uk

**Keywords:** microplastics, biodegradable mulch films (BDM), in vitro, lettuce, incubation

## Abstract

The use of biodegradable mulch films (BDM) in agriculture has raised concerns about the potential impact of the microplastics (MPs) they release over time, after the BDM’s useful life. The effects of BDM MPs have been explored through a diversity of assays, with still poorly understood and frequently contrasting results. Furthermore, the impact on plants as the MPs evolve in size and as a function of residence time in the soil remains largely unexplored. Through a controlled in vitro lettuce culture, this study explores the effect of BDM MPs size, using fractions 5 to <0.2 mm and pre-incubation times of 0 to 8 weeks, on plant development. Short incubation times, of 1 and 2 weeks, and freshly adding the BDM MPs inhibited plantlet growth, with smaller MPs inducing stronger effects. In contrast, longer MPs incubation, of 8 weeks, promoted plantlet development, enhancing leaf and particularly root elongation while reducing lateral root branching. The effects on roots were more pronounced, as the MPs size decreased. Germination and photosynthetic pigments were unaffected by any treatment. Overall, BDM MPs’ impact on plants was mainly driven by particle size and incubation time in the medium prior to seeding, with adverse effects on plant development observed at short incubation times that were no longer present when incubation was extended. These findings highlight the need to unravel the dynamic and temporal nature of the BDM MPs’ interaction with plants.

## 1. Introduction

The use of biodegradable mulches (BDMs) in agriculture is expanding due to the advantages that their biodegradability offers over conventional plastic mulches, which end up producing and accumulating microplastics (MPs; plastic fragments under 5 mm in size) into soils, polluting them [1]. The risks posed by conventional low-density polyethylene (LDPE) mulch MPs pollution have driven emerging interest in determining the safety and threats that BDM may pose to plants and soils before their complete biodegradation. As soon as BDMs begin to degrade in the environment and within the soil, they also generate MPs. Further fragmentation of BDM MPs, their contact with plant roots and the release of compounds that evolve into intermediates before full biodegradation is achieved may modify the agricultural environment [2].

Numerous studies conducted in pots and in the field have been published on the potential impact of BDM, with a variety of experimental conditions and cultivated species, both mono- and dicotyledonous, that exhibit different degrees of sensitivity [3,4], with lettuce standing out as one of the most sensitive for ecotoxicity studies [5]. Current estimates of the BDM MPs content in agricultural soils range from 0.01 to 1% (*w/w*) [3,6], with polybutylene adipate terephthalate (PBAT) or blends with thermoplastic starch (TP-starch) or with polylactic acid (PLA) being the most widely used and studied BDMs.

Results on plant growth and toxicity responses after exposure to mulch-derived MPs are frequently contrasting and controversial [7,8]. Comparisons between the effects of conventional and biodegradable mulches and their associated MPs on plants remain challenging due to the diversity of experimental designs [3], which results in data variability and hinders cross-comparison of findings [7]. In addition, while some studies analyze the effects of BDM on soils and plants under different environmental conditions [9], others assess their impacts through mesocosm studies with partially controlled experimental conditions (e.g., light, temperature or nutritional variations) [10]. Both approaches mimic the actual use of plastic mulches in the field, but the effects of residual MPs on plants are often masked by the specific soil and biological environment and by on-farm management interactions in field studies. Moreover, while the MPs’ size and residence time in soil prior to crop establishment are relevant determinants of their effects [10,11], they have been scarcely explored.

Previous studies have shown that in vitro experimental systems help to understand the specific effects of BDM MPs and their constituents on plant development, which are difficult to demonstrate within the complexity of in vivo studies [12]. In vitro culture allows specific evaluation of whole plants, simplifying the experimental system to a culture medium with a well-known chemical composition, where plants develop over time under fully controlled settings, without soil or environmental interactions. Furthermore, growing the plants in vitro facilitates analysis of the effects of different MPs types and sizes on plant development, and of the cocktail of compounds they may release and that subsequently evolve from them [13,14]. To contribute to filling the knowledge gap of the effects of BDM MPs on plants, this work aims to assess the influence of BDM MPs incubation time and size on lettuce development in vitro. We hypothesize that the impact of the BDM MPs on plants may be modulated by decreasing MPs size and increasing incubation time.

## 2. Results

### 2.1. One Week Incubation

This first assay was aimed at setting up a tissue culture system that would allow the investigation of whether the BDM MPs in the culture, at realistic concentrations, had effects on lettuce plantlet growth and development. The target was also to uncover if under the in vitro system the one-week pre-incubation of the MPs allows time for alterations in the release of compounds from the BDM MPs, as reported under other systems [13,15], was able to modify the plantlet response versus exposure to freshly dispersed MPs. The assay tested MPs of all sizes together (i.e., the sample was not fractionated but used as generated following sterilization) and malondialdehyde (MDA) was not determined in this assay, which was designed to validate the responsiveness of the system before assessing the size-specific effect of 0.1% concentration.

#### 2.1.1. Germination

The germination of lettuce seeds in the control treatment was first tested to guarantee that these reached the 70% threshold established by the OECD 208 Test Guideline [5], and germination was not affected by the treatments (control, 87.8%; MPs that were pre-incubated in the medium for 1 week, 97.5%; freshly dispersed MPs, 95.2%). Lettuce seeds showed no significant sensitivity to the presence of MPs in the medium, regardless of the incubation conditions (pre-incubation step or not).

#### 2.1.2. Morphophysiological Traits

The lettuce plantlets were grown for 4 weeks; then, plantlet height, root length, plantlet weight and photosynthetic pigments were determined, all of them being significantly affected by the presence of MPs, whether they were pre-incubated or freshly dispersed to the medium just before seeding.

**Figure 1:**
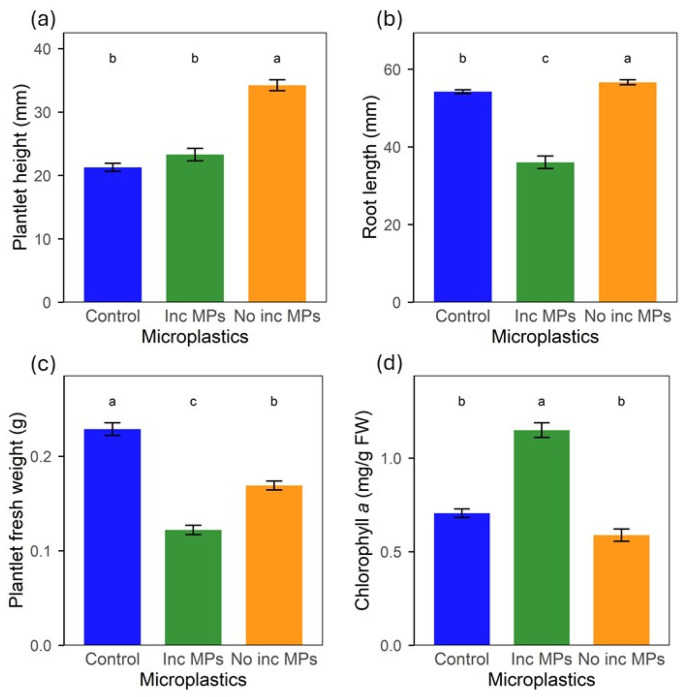
Lettuce plantlet development after 4 weeks growth in MS medium with BDM MPs pre-incubated for 1 week or freshly dispersed (not incubated); (**a**) plantlet height, (**b**) root length, (**c**) plantlet fresh weight, (**d**) chlorophyll *a* concentration. FW—Fresh weight. Inc MPs—Microplastics pre-incubated for 1 week before seeds were added. Not Inc MPs—No pre-incubation step; seeding just after freshly dispersing microplastics. Letters (a, b, c) show significant differences among treatments (*p* < 0.05). Bars show standard error.

The plantlets growing in the culture medium with freshly dispersed MPs (Not Inc MPs in the figure labels) were significantly taller compared to the untreated controls after 4 weeks, while MPs incubated for one week (Inc MPs in the figure labels) resulted in no significant effect on plantlet height but led to a marked decrease in root length compared to the untreated control (Figure 1a,b). Nevertheless, it was visually noticed that under both treatments the leaves were narrower than in the control plantlets, which likely accounts for the significant decrease in the plantlet fresh weight compared to the control. This was particularly pronounced when the MPs were pre-incubated in the medium for one week before the culture was started (Figure 1c).

The effects of the MPs exposure on photosynthetic pigments concentration in the leaves were opposite to those on plant weight and root development, with chlorophyll *a* (Figure 1d), chlorophyll *b*, and carotenoids (Figure S1) concentrations following the same pattern. Plantlets exposed to the pre-incubated MPs exhibited significantly higher photosynthetic pigment concentrations, with chlorophyll *a* increasing by 62% compared to the control and 71% for chlorophyll *b* and 46% for carotenoids, whereas exposure to freshly dispersed MPs to the medium resulted in 16%, 20% and 20% decreases relative to the control, respectively.

### 2.2. Two Week Incubation

After the first assay provided evidence that plant development was modified by the presence of MPs and that incubating the MPs in the medium modified their effects on plantlets, the aim of this experiment was to further explore the MPs’ effects on lettuce plantlets by extending the MPs’ pre-incubation time and fractionating the particles to assess size-specific effects. To assess whether the effects of the pre-incubation period stayed or scaled with its duration, the pre-incubation was extended 2 weeks, and to determine whether the MPs’ size was connected to their impacts three size fractions were utilized: 5–2, 2–0.2 and <0.2 mm.

#### 2.2.1. Germination

Lettuce seed germination in the control treatment was 84%, well over the 70% threshold established by the OECD 208 Test Guideline [5], and no significant effects were found for the treatments, although, as in the preliminary experiment, a trend towards higher germination rates was found in all MPs treatments (Table S1).

#### 2.2.2. Morphophysiological Traits

**Figure 2:**
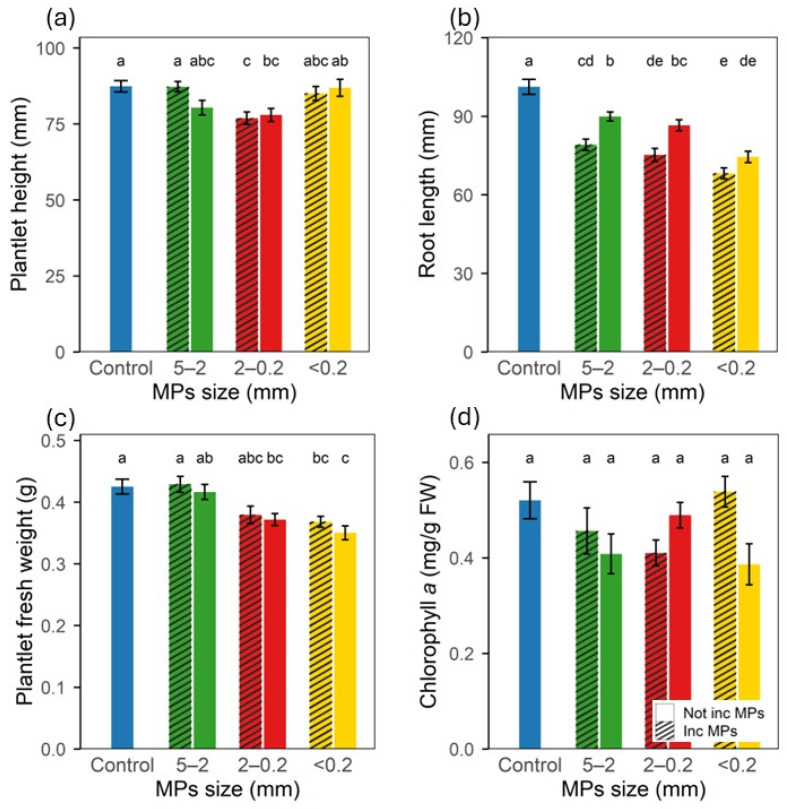
Lettuce plantlet development after 4 weeks growth in MS medium with BDM MPs size fractions pre-incubated for 2 weeks or not incubated; (**a**) plantlet height, (**b**) root length, (**c**) plantlet fresh weight. (**d**) chlorophyll *a* concentration. FW—Fresh weight. Inc MPs—Pre-incubated microplastics; Not Inc MPs—Seeding just after freshly dispersing microplastics. Letters (a, b, c, d, e) show significant differences among treatments (*p* < 0.05). Bars show standard error.

Plantlet height was overall not affected by the 2-week pre-incubation period of the MPs or by the MPs’ size, with shorter plantlets determined only for the 2–0.2 mm MPs (Figure 2a). On the contrary, all MPs significantly slowed down root elongation, and this effect was increasingly pronounced as the size of the MPs decreased, as well as being strengthened by the pre-incubation of the MPs; the highest reduction in the root length, 32.5%, was found for the <0.2 mm 2-week pre-incubated MPs treatment as compared to the control (Figure 2b).

The largest MPs fraction (5–2 mm) freshly dispersed or pre-incubated for 2 weeks in the medium had no significant impact on the plantlet weight, fresh or dry. However, when the plantlets were grown in the presence of smaller MPs fractions, both 2–0.2 mm and <0.2 mm, plantlet weight significantly decreased, with the magnitude of the effect increasing as the MPs’ size decreased (Figure 2c and Figure S2a).

Chlorophyll *a*, *b* and carotenoids concentrations in the leaves were not significantly decreased by the presence of MPs (Figure 2d and Figure S2b,c). However, the freshly dispersed MPs tended to decrease chlorophyll *a* and carotenoids concentrations more than the pre-incubated MPs in comparison to the non-exposed control plantlets. Lipid peroxidation, measured through MDA concentration, was not significantly altered by any of the MPs’ sizes (Figure S2d).

#### 2.2.3. Effects on the Medium

The variation in pH and conductivity after growing the plantlets for 4 weeks was very low, with the 5–2 mm non-incubated MPs as the only treatment resulting in slightly decreased pH and the <0.2 mm fraction causing slightly increased conductivity (Figure S3).

### 2.3. Eight Week Incubation

While a direct comparison between the 1- and 2-week pre-incubations is not possible, it is clear that the extended pre-incubation period of 2 weeks also results in modulation of the impacts of the BDM MPs on the plant development, particularly on roots, and that the magnitude of modulation varied across the MPs size fractions. Thus, the next set of experiments explored whether the effects of pre-incubation (simulating MPs aging) would persist if the MPs were pre-incubated for a longer period before cultivating the plantlets. Moreover, it was considered important to further resolve the intermediate effect of the 2–0.2 mm MPs by splitting this fraction into two (2–0.5 and 0.5–0.2 mm). Since the MPs’ most striking effects were on the roots, root development was regularly monitored, together with leaf development, from the first stages after germination. The not incubated MPs cultures were not included in this experiment, since their effects were examined in the 2-week incubation assay and no comparison to not incubated MPs is made. Thus, the pre-incubation time was extended to 8 weeks and the effects of four size fractions of BDM MPs (5–2; 2–0.5; 0.5–0.2 and <0.2 mm) were investigated.

#### 2.3.1. Effects on Germination

The germination of lettuce seeds in the control treatment was 91%, well over the 70% threshold established by the OECD 208 Test Guideline [5]. Globally, germination was very similar to the control treatment (Table S2).

#### 2.3.2. Effects During Plantlet Development

**Figure 3:**
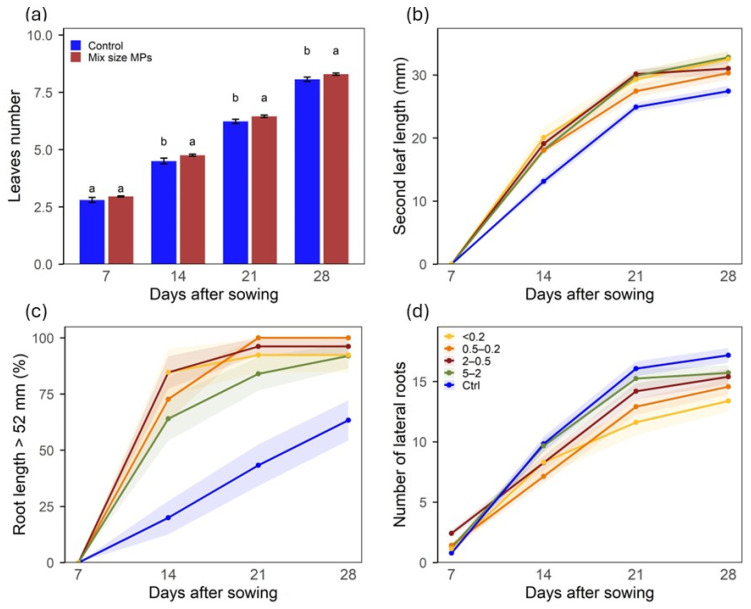
Lettuce plantlets over 4 weeks growth in MS medium with BDM MPs size fractions incubated for 8 weeks; (**a**) number of leaves with all sizes pooled together, (**b**) length of the second oldest leaf, (**c**) plantlets with roots reaching 52 mm (bottom of the culture container), (**d**) number of lateral roots. In panels (**b**–**d**) different colors are for the MPs sizes, and bars (**a**) or ribbons (**b**–**d**) for the standard error. Letters (a, b) show significant differences among treatments (*p* < 0.05).

Over the 4 weeks of growth, lettuce plantlet development differed between the control and MPs size fractions (Figure 3). As expected, linear mixed-effects models showed a strong effect of the time of culture (week, *p* < 0.001), reflecting normal plant development, without significant interaction effects between MPs size fractions and weeks of culture (Table 1). Hence, MPs’ effects were consistent throughout the growth period and were evaluated thereafter on a weekly basis using pairwise comparison.

Lettuce plantlets exposed to MPs, with size fractions pooled for this analysis to enable an overall comparison with the control, and pre-incubated in the medium for 8 weeks developed more leaves than control plantlets from the second week to the end of the culture (Figure 3a). Leaf growth followed the same pattern; the length of the second leaf, individually determined, was significantly higher for every week in plantlets exposed to MPs of any size compared with the control ones (Figure 3b), and MPs’ size had no significant effect on leaf length at any week of culture. The effects of the 8-week pre-incubation of MPs on the third leaf were similar for all size fractions (Figure S4a,b).

The in vitro culture facilitated time-course monitoring of the root system development, which was more substantially modified by the MPs’ presence than that of leaves. When exposed to the MPs, regardless of their size, the roots of the plantlets grew faster, with over half of the plantlets’ roots reaching at least 52 mm long significantly earlier that the roots of the control plantlets, from week 2 onwards (Figure 3c); concurrently, root branching declined as the size of the MPs decreased (Figure 3d).

#### 2.3.3. Morphophysiological Traits

**Figure 4:**
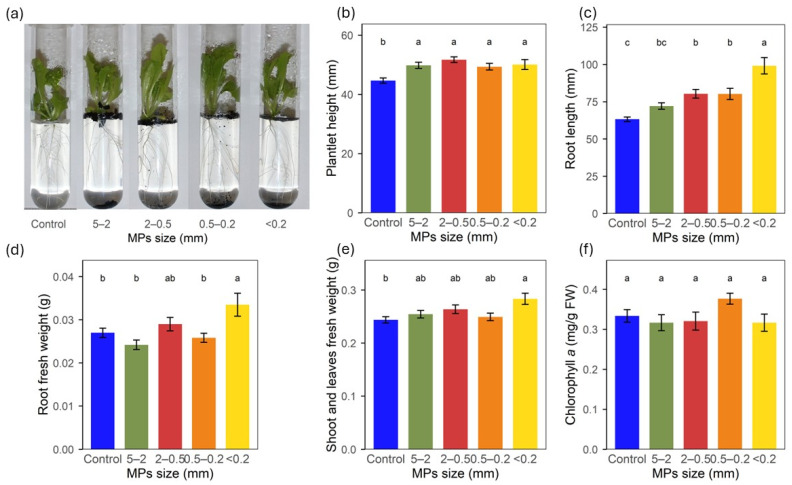
Lettuce plantlet development after 4 weeks growth in MS medium with BDM MPs size fractions incubated for 8 weeks; (**a**) visual images of plantlets development, (**b**) plantlet height, (**c**) root length, (**d**) root fresh weight, (**e**) shoot and leaves fresh weight, (**f**) chlorophyll *a* concentration. FW—Fresh weight. MPs—Microplastics. Letters (a, b, c) show significant differences among treatments (*p* < 0.05). Bars show standard error.

Visual effects of BDM MPs exposure on plantlets were not strikingly obvious by plain human observation (Figure 4a). However, in spite of no significant differences in the plantlet height, plantlets grown with MPs were taller than control ones (Figure 4b). On the roots, the effects were pronounced, with exposure to the MPs significantly increasing root length as the MPs size decreased; the smallest MPs, <0.2 mm size, resulted in 57% longer and 24% heavier roots (Figure 4c,d), while the shoot and leaves fresh weight increased by 16% (Figure 4e). The shoot and leaves dry weights were not affected by any of the treatments except in the roots grown with the smallest MPs which increased by 47% (Figure S5a,b). None of the BDM MPs treatments modified chlorophyll *a*, *b* and carotenoids contents (Figure 4f and Figure S5c,d). Lipid peroxidation, assessed by MDA concentration, was reduced by 22% with the 5–2 mm MPs treatment, despite a general non-significant trend of decreasing concentration in plants with decreasing MPs size (Figure S5e).

#### 2.3.4. Effects on the Medium

After 8 weeks of incubating the MPs, followed by 4 weeks of lettuce growth, the pH decreased slightly but significantly more in the medium with the MPs than in the control medium, and there was a trend to lower pH with decreasing MPs size. Conductivity was not affected by the incubation of the MPs (Figure S6).

## 3. Discussion

In this study, BDM MPs of different sizes were incubated directly in the in vitro culture medium for different durations prior to sowing the lettuce seeds (1, 2 and 8 weeks), a system that allowed monitoring of germination and lettuce development for 4 weeks. The observed differences on the one hand reflect the effect of the MPs size and on the other hand identify the potential variations associated with the leachates that the MPs are known to release over time.

### 3.1. Incubation Time Drives Plant Responses

Immediate cultivation and short incubation periods of BDM MPs, of 1 and 2 weeks, reduced lettuce biomass and root growth. The 1-week pre-incubation produced particularly strong growth inhibition, an effect likely linked to one or more of the compounds that have been reported to readily leach from PBAT BDM after incubation [12,13,15,16,17]. While the effects of these compounds have not been individually tested in the present in vitro system, several of them are reported to limit growth. For example, dibutyl-phthalate is released from PBAT BDM and limits corn plantlet growth [18]; tributyl acetyl citrate, also readily released from PBAT BDM, inhibits the growth of young lettuce seedlings [19]. The negative surface charge of PBAT MPs [20] facilitates interactions with the dissolved ions in the medium [21].

The physical presence of MPs alone is unlikely to explain the observed effects. Comparable growth inhibition, also with a strong reduction in root elongation, has been reported for lettuce plantlets when exposed to leachates from a 1-week incubation of BDM macro- and microplastics of the same composition [14], and when in a mesocosm pot system with PBAT MPs without prior incubation, both at 0.01 (*w/w*) [6]. Meanwhile, under a different experimental environment, the inhibition of plantlet development by PBAT MPs was limited unless at much higher concentrations (2% *w/w*) [22], highlighting the critical roles of the different experimental variables, among them the cultivation procedure, the size of the MPs and the unrevealed additives in the specific mulch formulations.

The BDM in this study has been demonstrated to readily leach a diversity of compounds from the first contact with a water solution [13], with peak concentrations frequently reached in the first 10 days and thereafter exhibiting decreasing dynamics from a few days to several months depending on their specific nature [16]; some of these compounds may contribute to the observed plant responses, themselves or through a cocktail effect. In contrast, the 8-week long incubation period alleviated or even reversed the MPs inhibitory effects produced at shorter incubation times: lettuce plantlets developed more and bigger leaves than control ones, longer roots, higher biomass and reduced lipid peroxidation. After incubation, the additives released by the BDM may undergo subsequent degradation and evolve over time into other compounds absent in the original mulch formulation. The results obtained suggest the potential of these compounds in enhancing plantlet development, or of exerting a cocktail effect. On the other hand, while no or short incubation times are indicative of the effects that the BDM MPs may cause on plants, the 8-week incubation time allows interactions of the MPs with the mineral solution of the culture medium for almost 2 months prior to the seed germination. This longer incubation may constitute a more realistic approach for evaluating the effects of the MPs in the natural environment, where they interact with the soil for weeks or months prior to interacting with seeds or plants [23].

Overall, the incubation timespan emerges as a key factor determining the impact of BDM MPs as stressors or stimulants for plant growth.

### 3.2. Size-Dependent Effects Are Modulated by Incubation Time

The size of the MPs was overall inversely correlated with their effects. Concurrently, the smaller the MPs were, the higher their specific surface area was, and thus the potential for release of compounds from the plastics surface [24]. This was particularly evidenced for root development. For the 2-week BDM incubation, the smallest MPs size (<0.2 mm) reduced root growth and biomass, suggesting enhanced chemical interference. In contrast, after the 8-week incubation prior to plantlet exposure, the smallest MPs were associated with enhanced root elongation and biomass. This shift from inhibitory effects under short pre-incubation times to compensatory or stimulatory responses under longer incubation times highlights the complexity of determining the MPs effects on plants: the smaller the MPs were, the higher the release rate of compounds was, with the smaller MPs usually exerting stronger negative effects on plant development, underscoring the importance of the MPs size on biological responses [25]. However, the nature of the compounds also determines their release and degradation dynamics. BDMs release a high diversity of additives to the soil solution. High amounts of many of them, and of the compounds evolved from them, have been generally observed for one month, but substantial quantities of many others remain for three months or even over half a year [16].

Hence, MPs’ size determines the potential magnitude of effects, whereas pre-incubation time determines their direction (inhibitory or stimulatory for growth).

### 3.3. Root System Is the Primary Target for MPs Effects

Despite being the first plant organ in direct contact with the soil environment, the root system has been poorly explored. Most BDM MPs studies have been focused mainly on germination, early growth and shoot-related endpoints [6,26,27,28], while studies on roots are most often limited to length [28,29] or biomass [29,30]. In vitro culture allows direct visual non-destructive monitoring of root development over time, including root architecture, a clear advantage as compared to pot or field studies. Consistent with roots as the primary target of underground interactions, direct exposure to MPs and their associated leachates within the culture medium resulted in roots responding strongly to the MPs and showing discrimination among treatments.

Following the 8-week pre-incubation, BDM MPs were associated with increased primary root elongation and reduced lateral root branching over the 4-week growth period. This pattern tended to be more pronounced as the MPs size decreased, enhancing the release of compounds from the increased reactive surface. Among the dozens of compounds known to leach from PBAT BDM [13,15], several are very close to endogenous plant compounds involved in plant growth regulation, such as butanediol, hydroxybutyric acid, or benzoic acid and its derivatives [31,32,33,34]. Glycerol and diacylglycerols particularly, proven to be released from the specific BDM tested and which at very low concentrations play a hormonal-related regulatory role and interact with auxins, are directly involved in changes in the architecture of the root system [35,36]. Testing these and other compounds released by the BDM, alone or in interaction in the in vitro system, would shed light on the hypothesis of their contribution to the observed reprogramming of root development.

Overall, BDM MPs reshape root architecture, with long MPs pre-incubation representing controlled pre-exposure conditions promoting root elongation while reducing lateral rooting.

### 3.4. Shoot and Leaf Development Shows Secondary Responses

While root development was limited from the 1-week incubation on, with clear size-dependent effects, the biomass of shoot and leaves was limited by the 1- and 2-week pre-incubation of the MPs but not by the 8-week pre-incubation. On the contrary, after the 8-week MPs incubation and regardless of the MPs size, the plantlet weight was similar to that of the control plantlets, suggesting a transient stress associated with the dynamics of the compounds leaching and degradation in the medium. Moreover, the 8-week MPs pre-incubation prompted lettuce plantlets to develop more and bigger leaves, in agreement with the likely enhanced absorbance of the leached compounds discussed above [15] through the enhanced root system. These results are consistent with others from studies performed under mesocosm systems, where BDM MPs induced only minor effects on lettuce plantlet biomass while triggering other physiological changes [3,26], stressing that the roots are more sensitive indicators to BDM MPs exposure than shoots and leaves [3,37].

The shoot and leaf responses occur after the changes in root development subsequent to the incubation of the MPs in the medium, rather than being direct effects of the BDM MPs themselves being toxic to the aerial tissues.

### 3.5. Overall Relevance and Implications of the Findings

Short-term exposure to BDM MPs misestimates the effects they may induce; in real life, the BDM MPs’ effects on plant development result from repeated incorporation into the agricultural environment, where they remain over extended periods (months to several years) and undergo physical and chemical transformations. In contrast, the long pre-incubation periods in the culture medium utilized here allow an extended interaction time closer to that occurring in the agricultural environment [23]. This study was conducted under in vitro conditions, which not only facilitates monitoring early growth as well as plant development over time, but, most relevantly, enabled detailed scrutiny of root system responses. While the in vitro system does not cover the complexity of agricultural soils, such as housing other organisms, it enables accurate comparisons by restricting the variability associated with the many agents interacting in these systems.

On the other hand, BDM MPs are not inert materials; they progressively break down into smaller and smaller fragments, releasing additive compounds during their aging and degradation which can alter plant and root development. Equally relevant, the effects on growth can persist even when classical markers do not report toxicity. This highlights the need to integrate MPs’ size and aging state into environmental risk assessment studies.

Further studies under soil-based and field conditions are required to evaluate the translation of the in vitro findings to the natural environment, allowing a comprehensive assessment of the long-term impacts of BDM MPs on agroecosystems.

## 4. Materials and Methods

### 4.1. Experimental Materials

A virgin, biodegradable black mulch film (certified according to EN17033:2018) [38] (Mater-Bi grade EF04P; Novamont SpA, Novara, Italy), 15 µm thick and 1.27 g/cm^3^ density, was used as the source for obtaining BDM MPs. The main composition of the material is a blend of PBAT, thermoplastic corn starch, and vegetable oils. The film was randomly cut into pieces (<20 cm^2^) with scissors and frozen with liquid nitrogen before being fed into a pre-cooled ultra-centrifugal mill (Retsch ZM200, Haan, Germany) with liquid nitrogen. When required by the corresponding experiment, the MPs were classified in 3 (5–2; 2–0.2 and <0.2 mm) or 4 (5–2; 2–0.5; 0.5–0.2 and <0.2 mm) size fractions using an electromagnetic vibrating sieve (FTS-0200, Filtra vibración, Badalona, Spain) fitted with metal sieves. All MPs were sterilized under a minimally invasive UV radiation [39] for 1 h.

### 4.2. Experiment Procedure

Tissue culture tubes were autoclave-sterilized and loaded with sterile MPs, 0.1% (*w/w*) under sterile conditions. Murashige and Skoog (MS) culture medium [40], with Gelrite^TM^ (DDBiolab, Barcelona, Spain) as gelling agent, was similarly autoclave-sterilized and thereafter added to the culture tubes with the MPs before solidifying. After running a preliminary test to ensure sterility of the medium-MPs system, 30 culture tubes for every treatment were considered for the experiments.

In the first pilot experiment, a set of the containers with the mix of MPs sizes was incubated for 1 week under the same environmental conditions (22 °C, 16 h light photoperiod, 230 µm·m^−2^·s^−1^ PAR) in which the trial was to be carried out (named Inc MP), while another set of containers freshly adding the mix of MPs sizes was prepared just before sowing of the seeds (called Not Inc MP). Experiments 2 and 3 followed the same procedure with some modifications: the MPs pre-incubation times were 2 weeks for Experiment 2 and 8 weeks for Experiment 3, and the MPs were distributed across different size ranges: 5–2, 2–0.2, and under 0.2 mm for Experiment 2, and 5–2, 2–0.5, 0.5–0.2, and under 0.2 mm for Experiment 3. Controls with only the MS medium were prepared similarly for all experiments.

Seeds of lettuce (*Lactuca sativa* L. cv. Batavia Grandes Lagos; Battle^®^, Molins de Rei, Spain) were surfaced sterilized by soaking with 2% sodium hypochlorite solution for 2 min. Three seeds per container were sown on the culture medium and germination was monitored regularly until stabilizing; then, only one seedling was retained. When occasional contamination occurred during the seed germination stage or during the incubation with MPs, the containers were immediately removed from the assays.

Plantlet development was monitored weekly to assess the overall growth and the plantlet height. Root length was monitored until they reached 52 mm in length, corresponding for most of the culture tubes to their bottom. At the end of the 28 days cultivation period, all plantlets were assessed for plantlet height and root length, and for fresh and dry weight. For all experiments and treatments, chlorophyll a, chlorophyll b, carotenoids and MDA contents were determined in leaf samples from 10 plantlets.

### 4.3. Determination of Fresh and Dry Weight

The plantlets were weighed right after being extracted from the culture medium, differentiating between the shoot and the root systems. After recording the fresh weights, the plantlets were dried in the oven for 4 days at 70 °C until reaching constant weight, and the dry weight and water content were determined.

### 4.4. Chlorophyll a, Chlorophyll b and Carotenoid Concentration

Samples ca. 12 mg weight from mature leaves were homogenized in 2 mL of cold 95% ethanol with a handheld homogenizer (D1000, Benchmark Scientific, Sayreville, NJ, USA) and centrifuged at 6000 rpm at 4 °C for 5 min. The supernatant absorbance was determined in duplicate with a microplate reader at 649, 665 and 470 nm. The concentration of chlorophyll (Chl) *a*, *b* and carotenoids (Ctn) (mg·g fresh weight) was determined using the Lichtenthaler equations [41]:

(1) [Chl a] = 13.95 × Abs(665) − 6.88 × Abs(649)

(2) [Chl b]=24.96 × Abs(649)−7.32 × Abs(665)

(3) $$\left[\mathrm{Ctn}\right]=\frac{\;1000\;\times \;\mathrm{Abs}\left(470\right)-2.05\;\times \;\left[\mathrm{chl}\;a\right]-114.8\;\times \;[\mathrm{chl}\;b]}{245}$$

### 4.5. Lipid Peroxidation

The lipid peroxidation, also referred to as the MDA content, was determined following the procedure of Hodges et al. 1999 [42], modified as described by Arbona et al. 2008 [43].

Disks from the youngest developed leaf, ca. 45 mg weight, were homogenized in 1 mL of 80% cold ethanol using a handheld homogenizer (D1000, Benchmark Scientific, Sayreville, NJ, USA) and centrifuged at 4500 rpm and 4 °C for 10 min. Half of the supernatant was added to Eppendorf tubes with 0.4 mL of 20% trichloroacetic acid (TCA) solution and the other half to 0.4 mL of 20% TCA + 0.6% thiobarbituric acid (TBA) solution. The Eppendorfs were incubated at 90 °C for 1 h in a thermoblock (QBD2, Grant Instruments, Royston, UK) before being placed in an ice bath. After a final centrifugation at 4500 rpm and 4 °C for 10 min, the absorbance was determined in duplicate with a microplate reader at 440, 534 and 600 nm. The concentration of MDA equivalents (nmol·g^−1^) was calculated as follows:

(4) $$A\;=\;\left[{\mathrm{Abs}\left(534\right)}_{+\mathrm{TBA}}\;-\;{\mathrm{Abs}\left(600\right)}_{+\mathrm{TBA}}\right]\;-\;[{\mathrm{Abs}\left(534\right)}_{-\mathrm{TBA}}\;-{\;\mathrm{Abs}\left(600\right)}_{-\mathrm{TBA}}]$$

(5) $$B=0.0571\;\times \;[{\mathrm{Abs}\left(440\right)}_{+\mathrm{TBA}}-{\mathrm{Abs}\left(600\right)}_{+\mathrm{TBA}}]$$

(6) $$\mathrm{MDA}\;\mathrm{equivalents}=[\frac{A-B}{157000}]\;\times {\;10}^{6}$$

### 4.6. Medium pH and Conductivity

At the end of the experiment, after plantlets had been taken out for the analyses described above, the tissue culture containers were frozen at −20 °C for facilitating later performance, and after defrosting their pH and conductivity were individually determined (SensION + MM374, Hach, Loveland, CO, USA).

### 4.7. Statistical Analysis

The in vitro experiments were carried out using a randomized complete block design. Statistical analyses were performed for every experiment using Rstudio software (2021.09.0). Differences among MPs treatments were evaluated through one-way analysis of variance (ANOVA), or through Welch’s ANOVA, or Kruskall–Wallis tests when required depending on normality of data and homogeneity of variances. Appropriate post hoc tests (Tukey HSD, Games–Howell, or Dunn’s test) were applied accordingly (α = 0.05). Comparisons between control plantlets and plantlets exposed to the different MPs treatments (pooled across size ranges, when appropriate) were performed using Student’s *t*-tests (α = 0.05).

For variables analyzed over time, linear mixed-effects models, with weeks included as a continuous variable, were carried out followed by a Holm post hoc test (α = 0.05) and a pairwise comparison within weeks. For the binary variable, whether roots reached the bottom of the tube or not, a generalized linear mixed-effects model with a binomial error distribution was applied, and Holm-adjusted pairwise comparisons within every week (α = 0.05) were performed.

## 5. Conclusions

This study evaluates how the particle size and pre-incubation time of BDM MPs affect lettuce development under a controlled in vitro culture system. Germination was not limited by any of the treatments. Nevertheless, plant growth responses were modulated by both MPs’ particle size and incubation time prior to sowing.

Short pre-incubation periods and freshly adding BDM MPs to the medium before seeding were associated with reduced plantlet biomass and the inhibition of root growth, with stronger effects observed for smaller MPs. However, after 8 weeks of pre-incubation of BDM MPs in the culture medium, their presence was related to enhanced primary root elongation and altered root architecture, while increasing plantlet biomass. The root system appeared as the primary and most sensitive target of BDM MPs exposure across experiments. These findings indicate that lettuce responses to BDM MPs are not static but depend on both particle size and the duration of their interaction with the surrounding medium prior to plant exposure.

The development of this in vitro system to assess the effects of BDM MPs on plantlets allowed close monitoring of plant development over time, particularly of roots, under controlled conditions. This approach facilitates discrimination of MPs size and incubation effects while minimizing environmental variability. However, the system does not represent the variability of soil environments. Further studies are required to better understand the size-dependent and incubation-dependent effects of BDM MPs in the complex agricultural system.

## Figures and Tables

**Table 1:** Results of linear mixed-effects models testing the effects of MPs size fraction, time (weeks), and their interaction on lettuce growth (number of leaves, 2nd leaf length, 3rd leaf length and root branching). Root length results come from a generalized linear mixed-effects model with a binomial error distribution. Statistically significant differences were determined based on alpha level *p* = 0.05. ns—non-significant.

Response Variable	Linear Mixed-Effects Model					
	Explanatory Variable				Interaction	
	MPs Size Fraction		Week		MPs Size Fractions × Week	
Endpoint	F (df_1_, df_2_)	*p*	F (df_1_, df_2_)	*p*	F (df_1_, df_2_)	*p*
Number of leaves	0.70 (4, 427.53)	ns	8146.80 (1, 422.53)	<0.001	0.31 (4, 422.53)	ns
2nd leaf length	2.98 (4, 310.39)	<0.05	416.15 (1, 308.15)	<0.001	0.89 (4, 308.15)	ns
3rd leaf length	0.55 (4, 338)	ns	1181.29 (1, 338)	<0.001	0.54 (4, 338)	ns
Root length	0.66 (4, 430.63)	ns	364.44 (1, 424.98)	<0.001	2.10 (4, 424.98)	ns
Root branching	1.08 (4, 302.09)	ns	220.39 (1, 300.55)	<0.001	0.86 (4, 300.55)	ns

## Data Availability

The original data presented in the study are openly available in [Zenodo] at [https://zenodo.org/communities/plasticunderground/records?q=&l=list&p=1&s=10&sort=newest, accessed on 25 November 2025]. Further inquiries can be directed to the corresponding author.

## Supplementary Materials

The following supporting information can be downloaded at https://www.mdpi.com/article/10.3390/plants15050849/s1, Figure S1: Lettuce plantlet development after 4 weeks growth in MS medium with BDM MPs pre-incubated for 1 week or freshly dispersed (not incubated); Table S1: Germination rate (%) of the lettuce plantlets after 10 days in MS medium with BDM MPs that were pre-incubated for 2 weeks or freshly prepared prior to seeding (not incubated); Figure S2: Lettuce plantlet development after 4 weeks growth in MS medium with BDM MPs size fractions pre-incubated for 2 weeks or freshly dispersed (not incubated); Figure S3: Culture medium pH, and conductivity after 4 weeks of lettuce growth with BDM MPs size fractions incubated for 2 weeks or freshly dispersed (not incubated); Table S2: Germination rate (%) of the lettuce plantlets after 10 days in MS medium with BDM MPs pre-incubated for 8 weeks; Figure S4: Length of the lettuce plantlet third leaf over 4 weeks growth in MS medium with BDM MPs pre-incubated for 8 weeks; Figure S5: Lettuce plantlets after 4 weeks growth in MS medium with BDM MPs size fractions pre-incubated for 8 weeks; Figure S6: Culture medium pH, and conductivity after 4 weeks of lettuce growth with BDM MPs size fractions pre-incubated for 8 weeks.

## Abbreviations

The following abbreviations are used in this manuscript:

BDM	Biodegradable mulch
LDPE	Low-density polyethylene
MDA	Malondialdehyde
MPs	Microplastics
MS medium	Murashige and Skoog culture medium
PBAT	Poly(butylene adipate-co-terephthalate)
PLA	Polylactic acid
TBA	Thiobarbituric acid
TCA	Trichloroacetic acid
TP-starch	Thermoplastic starch
