# Supplementary material for: Incubation Time and Size Effects of Biodegradable Mulch Microplastics on Lettuce Plantlets In Vitro
Source: Plants (Basel). 2026 Mar 9;15(5):849. doi: 10.3390/plants15050849 (PMC12986962; doi:10.3390/plants15050849)
Supplement: Supplementary file 1 [file plants-15-00849-s001.zip › plants-4162885-supplementary.pdf]

Supplementary material

For

Incubation Time and Size Effects of Biodegradable Mulch Microplastics on Lettuce Plantlets In Vitro

Mathilde Henrion, Lluis Martin-Closas, Iseult Lynch and Ana M. Pelacho

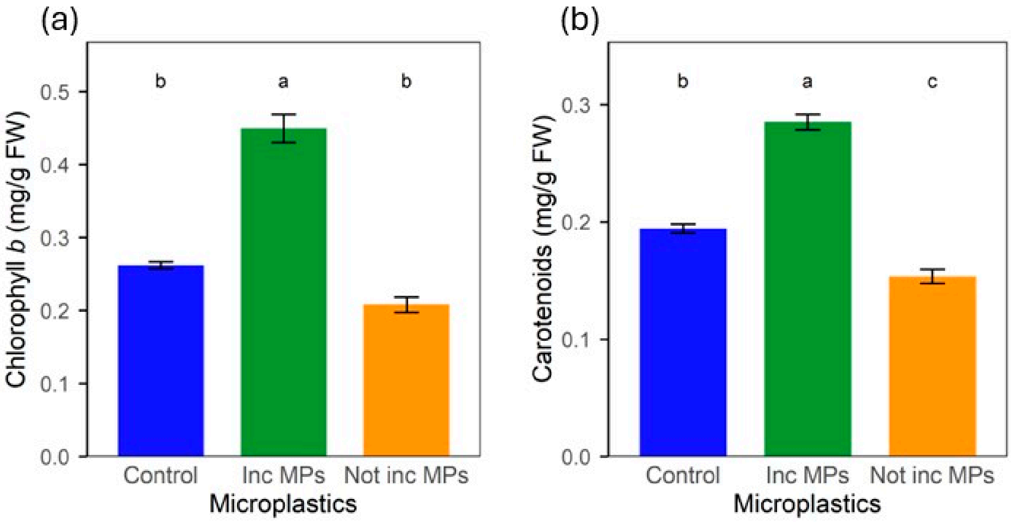

Figure S1. Lettuce plantlet development after 4 weeks growth in MS medium with BDM MPs (non-fractionated) pre-incubated for 1 week or freshly dispersed (not incubated); (a) chlorophyll *b* concentration, (b) carotenoids concentration. FW – Fresh weight; MPs – Microplastics; Inc – Incubated meaning that the MPs were pre-incubated in the medium for 1 week prior to seeding; Not inc – No incubation step, meaning that the MPs were dispersed immediately prior to addition of the seeds. Letters (a, b, c) show significant differences among treatments ($p < 0.05$). Bars show standard error. Control is plantlets grown in the MS medium only.

Table S1. Germination rate (%) of the lettuce plantlets after 10 days in MS medium with BDM MPs that were pre-incubated for 2 weeks or freshly prepared prior to seeding (no incubation).

Treatment	Control	Incubated MPs			No Incubation MPs		
MPs size (mm)		5-2	2-0.2	<0.2	5-2	2-0.2	<0.2
Germination (%)	84.2	92.8	91.6	89.1	90.0	93.3	90.0

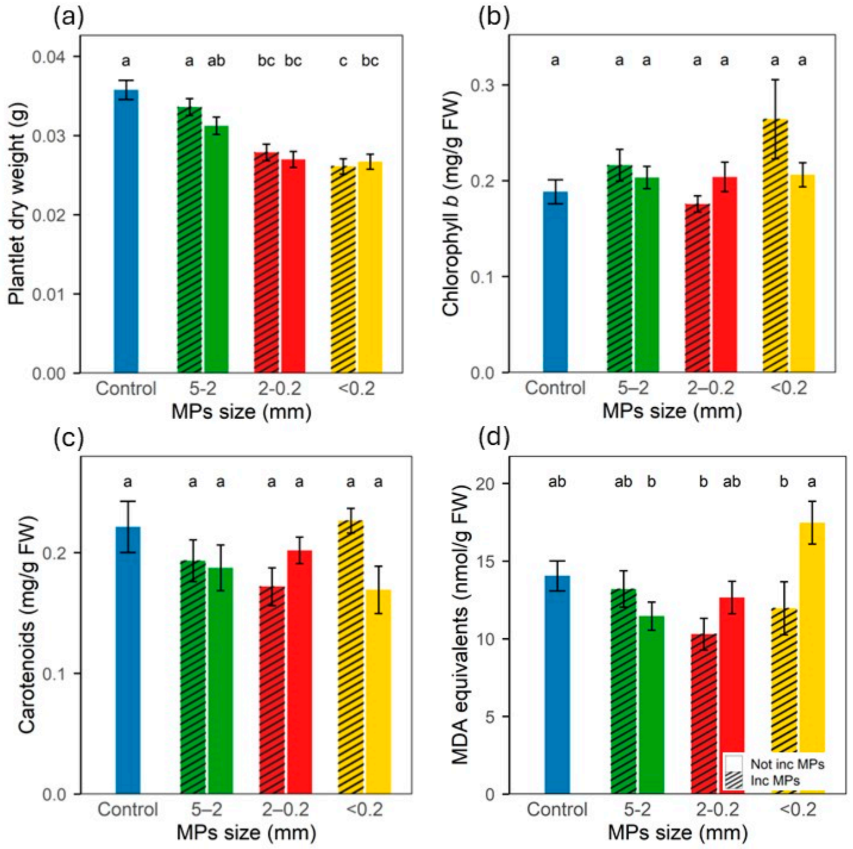

Figure S2. Lettuce plantlet development after 4 weeks of growth in MS medium with BDM MPs size fractions pre-incubated in the medium for 2 weeks or freshly dispersed (not incubated); (a) plantlet dry weight, (b) chlorophyll *b* concentration, (c) carotenoids concentration, (d) MDA equivalents. FW – Fresh weight; MPs – Microplastics; Inc – Incubated; Not inc – No incubation step meaning that the MPs were dispersed immediately prior to addition of the seeds. Letters (a, b, c) show significant differences among treatments ($p < 0.05$). Bars show standard error.

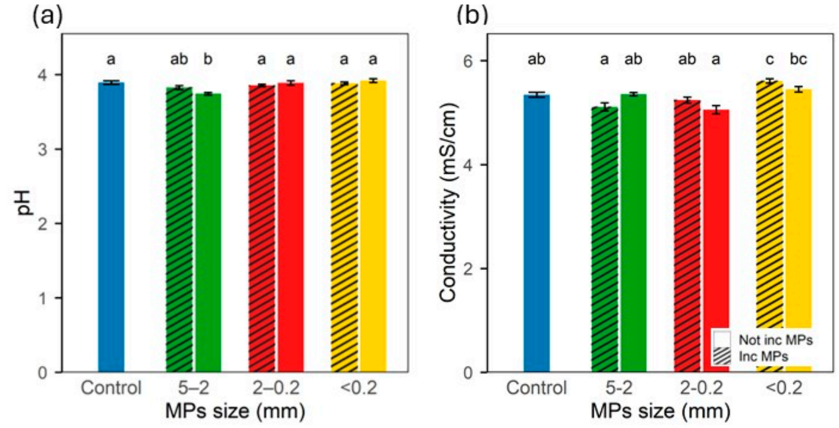

Figure S3. Culture medium (a) pH, and (b) conductivity after 4 weeks of lettuce growth with BDM MPs size fractions pre-incubated for 2 weeks or not incubated, meaning that the MPs were dispersed immediately prior to addition of the seeds, compared to the untreated control. Letters (a, b, c) show significant differences among treatments ($p < 0.05$). Bars show standard error.

Table S2. Germination rate (%) of the lettuce plantlets after 10 days in MS medium with BDM MPs pre-incubated for 8 weeks.

**MPs size (mm)**	**Control**	**5-2**	**2-0.5**	**0.5-0.2**	**<0.2**
Germination (%)	91.1	89.3	98.8	74.4	91.2

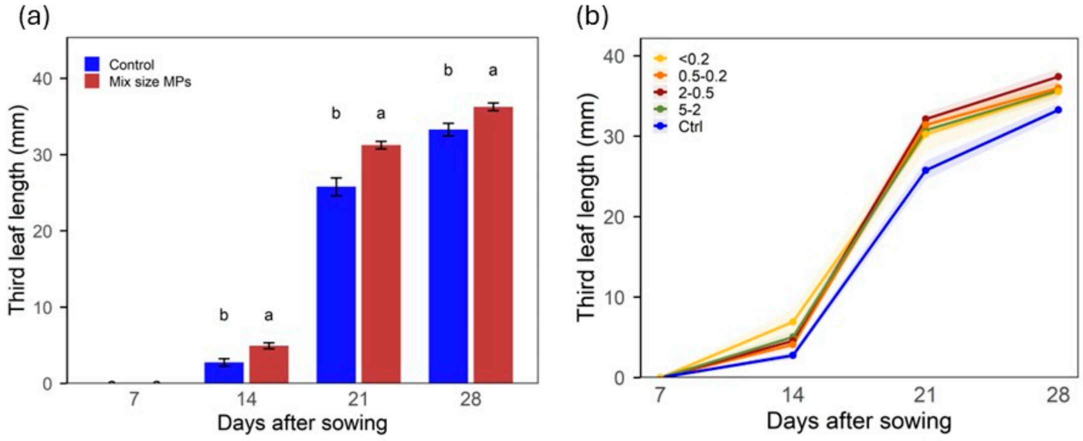

Figure S4. Length of the lettuce plantlet third leaf over 4 weeks growth in MS medium (a) pooling all sizes together; (b) differentiating size fractions of BDM MPs pre-incubated for 8 weeks. MPs - Microplastics. Letters (a, b) show significant differences among treatments ($p < 0.05$). Bars show standard error.

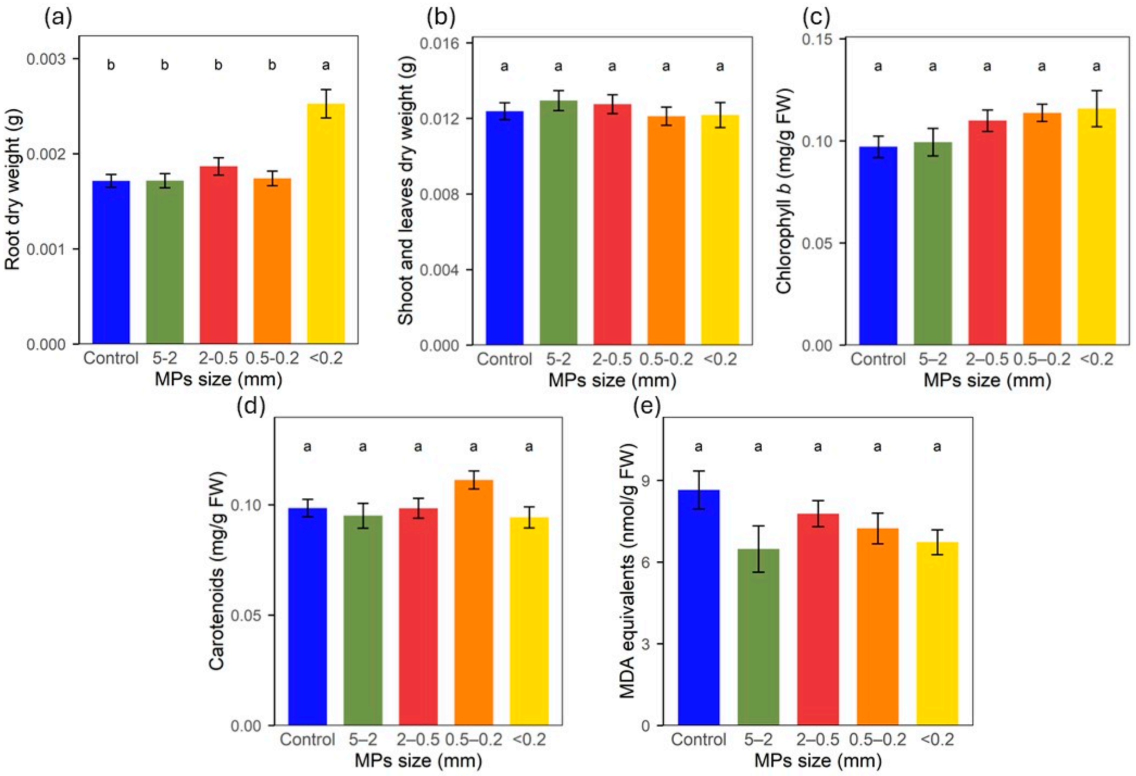

Figure S5. Lettuce plantlets after 4 weeks growth in MS medium with BDM MPs size fractions pre-incubated for 8 weeks; (a) root dry weight, (b) plantlet dry weight, (c) chlorophyll *b* concentration, (d) carotenoids concentration, (e) MDA equivalents. MPs – Microplastics. Letters (a, b) show significant differences among treatments ($p < 0.05$). Bars show standard error.

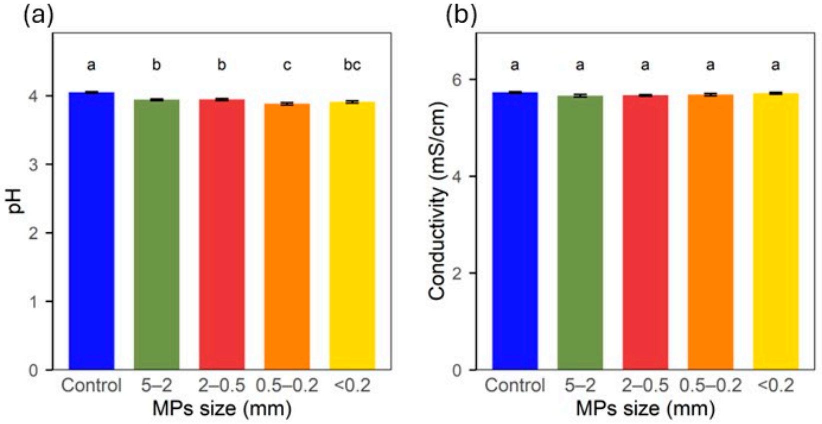

Figure S6. (a) pH, (b) conductivity of the medium after 4 weeks of lettuce growth with BDM MPs size fractions pre-incubated for 8 weeks. Letters (a, b) show significant differences among treatments ($p < 0.05$). Bars show standard error.
